# Risk Factors for Gallstone Disease in a Thai Population

**DOI:** 10.2188/jea.JE20080019

**Published:** 2009-05-05

**Authors:** Sukij Panpimanmas, Charuwan Manmee

**Affiliations:** 1 Department of Surgery, Rajavithi Hospital, College of Medicine, Rangsit University, Bangkok, Thailand; 2 Department of Academic Support, Rajavithi Hospital, Bangkok, Thailand

**Keywords:** gallstone disease, risk factor, Thai population

## Abstract

**Background:**

Gallstone disease (GSD) is a major public health problem that is associated with a number of risk factors.

**Methods:**

We conducted a case–control study of 407 participants comprising 207 cases with GSD and 200 controls without GSD, as confirmed by ultrasonography. The participants completed a questionnaire and underwent physical and ultrasonographic examination. The risk factors examined were age, sex, BMI, use of oral contraceptives, diabetes mellitus, cirrhosis, thalassemia, dyspepsia, family history of gallstone disease, smoking status, alcohol consumption, and dietary history.

**Results:**

BMI, fat content of dietary meat, and smoking were associated with GSD. When compared to participants with a BMI below 25, participants with a BMI of 25 or higher had a multivariate relative risk of 4.1 (95% CI, 2.5–6.7). Participants who consumed meat with moderate fat content or high fat content had respective relative risks of 2.5 and 2.9 (95% CI, 1.5–4.2 and 1.5–5.6), when compared to those who consumed meat with a low fat content. Also, the multivariate relative risk for former smokers, as compared to never smokers, was 2.4 (95% CI, 1.1–5.2).

**Conclusions:**

High BMI, consumption of high-fat meat, and smoking were associated with gallstone disease.

## INTRODUCTION

Gallstone disease (GSD) is a major public health problem in many countries, and is a significant cause of morbidity in the United States. More than 500 000 cholecystectomies are performed yearly, and the total direct cost for the diagnosis and treatment of gallstones is conservatively estimated at $5 billion annually.^1^ In Sweden and Czechoslovakia, the autopsy incidence of gallstones in individuals older than 20 years was 30% in men and 50% in women.^2^ In addition, statistics from the Ministry of Public Health in Thailand show that the numbers of new inpatient cases of gallstones from 1999 to 2005 were 23 105, 24 438, 24 472, 30 754, 35 895, 41 019, and 48 232 cases. GSD is the most common disease of the biliary system and most cases are asymptomatic.^3^ Therefore, given the magnitude of the problem, strategies to reduce the incidence of GSD are vital.

A review of the prevalence and risk factors for GSD suggests that lifestyle modifications may reduce the risk for gallstones. Many previous studies have shown that the risk factors for gallstones are multifactorial. The risk factors associated with GSD are age, race, sex, family history of GSD, obesity, rapid weight loss, number of children, and use of oral contraceptives or estrogen replacement therapy.^4^ In a study of gallstones in a Danish population, it was reported that BMI and slimming treatments were significantly associated with GSD.^5^ In Taiwan, age over 60 years and diabetes mellitus were risk factors for GSD among older persons.^6^ Although there have been a number of studies on the factors associated with gallstone formation in recent years, the reasons for the rising incidence of gallstones are unknown.^7^ A clearer understanding of the risk factors may help us to identify patients with GSD and to reduce the risk of GSD in some patients. Therefore, we conducted a case–control study to identify risk factors associated with GSD in the Thai population.

## PARTICIPANTS AND METHODS

### Population and sampling

The study comprised 407 participants enrolled between January 2005 and June 2006 at the Department of Surgery at Rajavithi Hospital and included outpatients. The case participants comprised 207 consecutive patients with a recent diagnosis of GSD that had been confirmed by ultrasonography. The control participants comprised 200 consecutive asymptomatic outpatients who sought treatment at our hospital for conditions other than GSD. The study was performed after approval was granted by the Ethics Committee of Rajavithi Hospital and informed consent was obtained from all participants. The demographic characteristics of the cases and controls are shown in Table 1.

**Table 1.** Demographic characteristics of cases and controls

Characteristics	Cases *n* (%)		Controls *n* (%)		*P* value
1. Sex					0.120
​ Male	75	(36.2)	58	(29.0)	
​ Female	132	(63.8)	142	(71.0)	
2. Age					<0.001^*^
​ ≤39	27	(13.0)	58	(29.0)	
​ 40–60	100	(48.3)	101	(50.5)	
​ >60	80	(38.7)	41	(20.5)	
​ Mean (SD)	56	(14.3)	48	(14.3)	
3. Nationality					0.144
​ Thai	196	(94.7)	195	(97.5)	
​ other	11	(5.3)	5	(2.5)	
4. Occupation					0.060
​ salaried worker	54	(26.2)	65	(32.5)	
​ civil servant	45	(21.8)	55	(27.5)	
​ merchant	27	(13.1)	27	(13.5)	
​ other	81	(38.9)	53	(26.5)	
5. Income (Thai baht/month)					0.836
​ <5500	80	(38.6)	76	(38.0)	
​ 5501–10 000	54	(26.1)	46	(23.0)	
​ 10 001–20 000	34	(16.4)	42	(21.0)	
​ 20 001–30 000	21	(10.2)	20	(10.0)	
​ >30 000	14	(6.8)	13	(6.5)	
6. BMI					<0.001^†^
​ ≤24.9	102	(49.5)	160	(80.4)	
​ ≥25	104	(50.5)	39	(19.6)	
​ Mean (SD)	25.4	(5.1)	22.8	(4.4)	

*Pearson’s Chi-square.

^†^Mann–Whitney test.

### Data collection

Each participant selected for the study was personally interviewed in the outpatient department and the ultrasound room. Although clinical examinations were conducted during the day, ultrasonographic examinations were conducted during and outside normal examination hours. Interviews were conducted by trained and supervised interviewers: 1 physician and 1 nurse. The response rate was approximately 95% among both cases and controls. Participants with GSD were examined by ultrasonography to confirm the diagnosis. Cases were defined as participants with clinical GSD. Participants with no evidence of gallstones on ultrasonography were classified as controls.

During the interview, all participants were asked to provide information on sex, age, past medical history (number of children, years of oral contraceptive use), and other demographic data. They were also asked to describe their food preferences, frequency of consuming high-fat foods, the fat content of the meat they consumed, and the parts of poultry that they consumed. The presence or absence of cirrhosis and history of diabetes mellitus were also determined by reviewing participants’ medical records and symptoms. Participants with diabetes were asked about the date and their age at diagnosis. Height without shoes and weight while clothed were recorded. Body mass index (BMI) (kg/m^2^) was used as a measurement of obesity. Normal weight was defined as a BMI less than 25; those with a BMI of 25 or more were classified as overweight or obese. Ever smokers were classified as former smokers or current smokers, and their exposure level was noted. Ever drinkers were classified as former drinkers or current drinkers, and the duration and frequency of alcohol consumption was recorded.

### Statistical analysis

Data from the questionnaires were analyzed using SPSS version 11.5. The demographic data were expressed as mean ± standard deviation (SD). Differences in demographic characteristics of participants were compared by using the Mann–Whitney test for continuous variables (age and BMI) and Pearson’s chi-square test for categorical variables (sex, nationality, occupation, and income). Multivariate analysis by unconditional binary logistic regression was used to determine odds ratios and 95% CIs for the risk of GSD (adjusted for age and sex).

## RESULTS

The demographic characteristics of the participants are shown in Table 1. Among both cases and controls, more than 60% of the participants were women.

The average age of the cases was 56 (range, 14–84), which was significantly higher than that of the controls (48 years; range, 12–90). The participants were subdivided into 3 age groups; approximately 50% of cases and controls were aged between 40 and 60. Among both groups, nearly all participants were Thai nationals. The most common occupation among cases was “other”; “salaried worker” was the most common occupation among controls. Approximately 40% of participants reported earning less than 5500 Thai baht per month. Most cases had a BMI of 25 or higher, while most controls had a BMI of 24.9 or lower. The average BMI (± SD) among the case participants was 25.4 (± 5.1), which was significantly higher than that of the control participants (22.8 ± 4.4). Only age and BMI significantly differed between cases and controls (*P* < 0.001).

On univariate analysis, age over 30 years, high BMI, history of smoking, a history of alcohol consumption, a diet high in calories, and a diet high in animal fat and protein, were positively correlated with the prevalence of gallstones.

Table 2 shows the results of multivariate analysis of factors associated with GSD using unconditional multiple logistic regression. The multivariate factors, adjusted for both age and sex, were BMI, history of smoking, period of alcohol consumption, fat content in dietary meat, and diabetes status. The results showed that only BMI, history of smoking, and fat content in dietary meat were significantly associated with GSD. Participants with a BMI of 25 or higher had a multivariate relative risk of 4.1 (95% CI, 2.5–6.7), as compared to participants with a BMI lower than 25.

**Table 2.** Risk factors associated with gallstone disease

Factor	Comparison	OR (95% CI)*	OR (95% CI)^†^	OR (95% CI)^‡^
1. BMI	≥25 vs. ≤24.9	4.0 (2.5–6.3)	4.1 (2.5–6.7)	4 (2.5–6.6)
2. Smoking history	Former smoker vs. Never smoker	2.3 (1.2–4.5)	2.4 (1.1–5.2)	2.2 (1.0–4.8)
	Current smoker vs. Never smoker	1.5 (0.7–3.6)	1.8 (0.7–4.6)	1.6 (0.6–4.5)
3. Period of alcohol consumption	<5 years vs. Nondrinker	1.1 (0.5–2.2)	1.0 (0.5–2.2)	1.0 (0.4–2.2)
	5–10 years vs. Nondrinker	1.7 (0.9–3.2)	1.2 (0.6–2.4)	1.2 (0.6–2.5)
	>10 years vs. Nondrinker	0.9 (0.4–1.9)	0.8 (0.4–1.9)	0.9 (0.4–2.2)
4. Fat content in dietary meat	Moderate vs. Low	2.4 (1.5–3.9)	2.5 (1.5–4.2)	2.1 (1.2–3.5)
	High vs. Low	3.0 (1.6–5.5)	2.9 (1.5–5.6)	3.4 (1.7–7.0)
5. Diabetes Mellitus	Yes vs. No	2.0 (1.0–4.2)	1.6 (0.7–3.4)	1.6 (0.7–3.5)

*Adjusted only for age and sex by unconditional binary logistic regression.

^†^Multivariate adjustment, plus age and sex adjustment, by logistic regression.

^‡^Multivariate adjustment, plus age and sex adjustment, by logistic regression (participants older than 30 years).

A history of smoking remained significantly associated with GSD, and the trend effect was significant among former smokers. As compared to never smokers, former smokers had a multivariate relative risk of 2.4 (95% CI, 1.1–5.2).

The period of alcohol consumption was not a significant risk factor for GSD, as there were no significant differences among participants who had never consumed alcohol and those who had consumed alcohol for less than 5 years, between 5 and 10 years, or more than 10 years. In addition, a diagnosis of diabetes mellitus was not associated with GSD.

Regarding diet history, the fat content in dietary meat was associated with GSD (*P* < 0.05). The composition of diets (ie, a diet high in calories, animal fat and/or protein) was positively correlated with a higher prevalence of gallstones. Participants who consumed meat with a high fat content or moderate fat content had a multivariate relative risk of GSD of 2.9 (95% CI, 1.5–5.6) and 2.5 (95% CI, 1.5–4.2), respectively, as compared to participants who consumed meat with a low fat content.

Multivariate logistic regression analysis, adjusted for age and sex, confirmed the results of univariate analysis: BMI, a history of smoking, and fat content of dietary meat were positively associated with GSD. In addition, the odds ratios for these risk factors were similar in these analyses.

Odds ratios were also calculated for GSD risk factors, including smoking status and alcohol consumption, among participants older than 30 years. BMI and fat content of dietary meat remained associated with GSD among this subgroup, as did a history of smoking. The odds ratio for GSD among former smokers older than 30 was similar to that among all participants.

Many previous studies reported that GSD is associated with specific factors, such as number of children and oral contraceptive use, among women. These factors were also investigated in the present study and the results are shown in Table 3. Only BMI and fat content of dietary meat were associated with GSD in women. These results were confirmed in multivariate logistic regression analysis adjusted for age, which showed that females with a BMI of 25 or higher had a multivariate relative risk for GSD of 5.2 (95% CI, 2.8–9.7), when compared to females with a BMI lower than 25. This odds ratio was nearly identical to that calculated after adjusting for age only (5.3). In addition, the fat content of dietary meat was associated with GSD in females (*P* < 0.05). When compared with females who consumed low-fat meat, females who consumed meat with a high fat content or moderate fat content had a multivariate relative risk for GSD of 2.7 (95% CI, 1.1–6.3) and 2.3 (95% CI, 1.2–4.4), respectively. The period of alcohol consumption was not associated with GSD in females in any of the analyses. Also, there was no association with diabetes status, number of children, or duration of contraceptive use. In women older than 30 years, only BMI and consumption of high-fat meat were associated with GSD (*P* < 0.05). Period of alcohol consumption and duration of contraceptive use may be related to GSD, especially for people aged above 30 years. Although women older than 30 with experience of alcohol consumption between 5 and 10 years would have odds ratio of 2.2 compared with females in all age groups (OR = 1.9), there was no statistically significant risk between this factor and GSD.

**Table 3.** Risk factors associated with gallstone disease in females

Factor	Comparison	OR (95% CI)*	OR (95% CI)^†^	OR (95% CI)^‡^

1. BMI	≥25 vs. ≤24.9	5.3 (3.0–9.5)	5.2 (2.8–9.7)	5.4 (2.9–10.2)
2. Period of alcohol consumption	<5 years vs. Nondrinker	1.1 (0.4–2.6)	1.1 (0.4–3.1)	0.9 (0.3–2.6)
	5–10 years vs. Nondrinker	2.0 (0.8–5.2)	1.9 (0.6–5.7)	2.2 (0.7–7.2)
	>10 years vs. Nondrinker	0.8 (0.4–2.0)	1.0 (0.4–2.6)	1.1 (0.4–3.1)
3. Fat content in dietary meat	Moderate vs. Low	2.2 (1.2–3.7)	2.3 (1.2–4.4)	1.8 (0.9–3.5)
	High vs. Low	2.6 (1.2–5.6)	2.7 (1.1–6.3)	2.9 (1.2–7.2)
4. Diabetes Mellitus	Yes vs. No	2.6 (1.1–6.1)	1.8 (0.7–4.7)	1.8 (0.7–4.8)
5. Number of children	1–2 vs. 0	2.7 (1.3–5.3)	1.3 (0.7–2.6)	1.3 (0.7–2.7)
	≥3 vs. 0	3.7 (1.8–7.7)	0.5 (0.2–1.1)	0.5 (0.2–1.2)
6. Duration of Contraceptive use	<5 years vs. never	1.3 (0.7–2.4)	1.2 (0.6–2.6)	1.2 (0.5–2.5)
	5–10 years vs. never	1.1 (0.5–2.6)	0.6 (0.2–1.6)	0.4 (0.2–1.3)
	>10 years vs. never	1.9 (0.5–7.4)	0.7 (0.2–3.1)	0.7 (0.2–3.0)

*Adjusted only for age by unconditional binary logistic regression.

^†^Multivariate adjustment, plus age adjustment, by logistic regression.

^‡^Multivariate adjustment, plus age adjustment, by logistic regression (participants older than 30 years).

## DISCUSSION

We compared individuals with and without ultrasonographic evidence of gallstones. The numbers of participants in each group were almost equal and participants were nearly all consecutively enrolled in the study. The findings of the present study confirm and expand upon those of previous studies investigating the importance of BMI,^8^ a history of smoking,^5^ and consumption of high-fat meat^9^ in the epidemiology of GSD.

Gallstones can occur at any age but are unusual in individuals younger than 30 years. There is evidence that gallstones progressively increase with age. For instance, in a study using ultrasonographic population screening, the prevalence of gallstones in Mexican-Americans, Cuban-Americans, and mainland Puerto Ricans increased with age and was higher in women especially the Mexican-Americans women age 60–74 yr had prevalence reach to 44.1%.^10^ In addition, a study of senior citizens in Taiwan^6^ found that age over 60 years was the most important risk for the development of gallstones. Also, gallstones were significantly more frequent in Jewish residents of Tel Aviv than in Arab residents of Gaza.^11^ This difference was entirely due to the higher prevalence of gallstones in patients older than 60 years; the difference was minimal or absent in patients aged 40 to 59 years. However, the association between age and gallstones was not significant, which suggests that age is a confounder, due to its correlation with other salient factors. Many studies have observed a higher prevalence of gallstones in women, which was also noted in the present study. However, we believe that this may result from selection bias resulting from a highly unequal female-to-male ratio. Nationality, occupation, and income did not significantly differ between cases and controls.

In the present study, BMI was positively associated with GSD. When participants were divided into those with a BMI lower than 25 and those with a BMI of 25 or higher, the odds ratios for all overweight participants and that for overweight participants aged over 30 years were very similar (4.1 and 4.0, respectively). This finding is in agreement with the results from several studies of clinically diagnosed gallstones. A number of reports suggest that the relative risk for GSD is markedly higher for the most obese individuals.^1^ Among women, BMI has been identified as a risk factor for gallstones. Obese individuals are exposed to higher levels of biliary secretion of cholesterol from the liver, which results in the production of bile supersaturated with cholesterol and induces precipitation of monohydrate cholesterol microcrystals that grow, agglomerate, and form gallstones.^12^ In addition, overweight increases the chance of developing a number diseases other than GSD.

Consumption of high-fat foods is associated with a higher risk for GSD. Nunes and Beckingham^13^ observed that consumption of saturated fats and refined sugar is associated with GSD and that gallstone formation increases with a rise in daily calorie intake. Furthermore, Misciagna et al^9^ observed that a diet high in fat was associated with a higher risk for GSD. In addition, a study in India found that consumption of tamarind, which is common in the Southern Indian diet, is a risk factor for gallstone formation.^14^ We hypothesize that a diet high in fat results in cholesterol saturation of bile or cholelithiasis.^15^ A high total fat intake is also associated with an increased risk for gallstones; therefore, gallstone prevalence is lower in vegetarians.^1^ Although dietary factors may appear to be associated with gallstones, the link between diet and gallstone formation is not be straightforward. For example, it is possible that individuals may limit fatty food consumption in response to gallstone-related symptoms, which could lead to a reduction in the incidence of GSD, ie, diet may not be a risk factor for GSD.

We noted that a history of smoking was a risk factor for gallstones, particularly so in a comparison of former smokers and never smokers. With never smokers as the referent, the odds ratio for former smokers was 2.4, which was slightly higher than the odds ratio of 2.2 for former smokers aged over 30 years; however, there was no significant association between the prevalence of gallstones and smoking status in the participants aged over 30 years. This may be because most former smokers in this study were younger than 30 years. It seems that advanced age may be associated with higher gallstone prevalence among these participants; however, the association was not statistically significant. The findings of the present study agree with those of a previous study conducted in Denmark,^5^ which found that smoking was related to gallstones in men, but not in women.

The period of alcohol consumption was not a risk factor of gallstones, which is in agreement with the findings from a study of the Hispanic population of the United States,^16^ in which alcohol consumption was negatively associated with GSD.

Previous studies indicated that cirrhosis is a risk factor for GSD, and cirrhosis and chronic hemolytic disease have been shown to increase the risk for pigmented stones.^17^^,^^18^ In the present study cirrhosis was not associated with gallstones in binary logistic regression analysis. However, it should be noted that only 2 participants with cirrhosis met the diagnostic criteria.

We noted no significant association between diabetes status and gallstones, which confirms the findings of Jorgensen et al,^5^ who observed no association between DM and gallstones in a Danish cross-sectional study of 4581 individuals. However, earlier studies have reported conflicting results.

This study showed that higher parity was not associated with GSD. However, Scragg et al^19^ noted an increased risk for gallstones during pregnancy. The risk was highest for participants younger than 29 years, an observation that has been confirmed by other studies. Scragg et al^19^ also observed that pregnancy was significantly associated with an increased risk for gallstones among younger women, but not among postmenopausal women older than 50 years. Stone formation during pregnancy may be due to sluggish contractility associated with lithogenic bile induced by endogenous estrogens.^1^

Among women, use of oral contraceptives was not a risk factor for gallstones. However, this may have resulted from recall bias. The questionnaire used in the present study requested information only on the duration of contraceptive use; it did not request information on the type or dose of contraceptives. Hence, we believe that caution is warranted in interpreting this finding.

It is important to note that because the present study was an unmatched case–control study, the demographic characteristics of the participant groups may substantially differ. If we had elected to match cases and controls, the results would have been more conclusive, but the time required would have been prohibitive. Secondly, the number of participants with cirrhosis was very small (only 2). Thirdly, the possibility of selection bias must be acknowledged. Finally, thalassemia and dyspepsia were uncommon among our participants.

In conclusion, our findings indicate that a high BMI and consumption of high-fat food may increase the risk for GSD in Thais. In addition, a history of smoking was associated with gallstones, particularly in males. Age and sex were not risk factors, although this may have been due to selection bias.
